# Effect of different cell culture media on the production and glycosylation of a monoclonal antibody from a CHO cell line

**DOI:** 10.1007/s10616-025-00733-7

**Published:** 2025-03-22

**Authors:** Jaeweon Lee, Uriel Ortega-Rodriguez, Chikkathur N. Madhavarao, Tongzhong Ju, Thomas O’Connor, Muhammad Ashraf, Seongkyu Yoon

**Affiliations:** 1 Office of Pharmaceutical Quality Research, CDER, U.S. FDA, 10903 New Hampshire Ave, Silver Spring, MD 20993 USA; 2 Department of Chemical Engineering, University of Massachusetts Lowell, Lowell, MA USA

**Keywords:** Chemically defined medium, Monoclonal antibody (mAb), Titer, Glycosylation, N-glycans

## Abstract

Recombinant monoclonal antibodies (mAbs) are commonly produced using Chinese hamster ovary (CHO) cells and the cell culture medium used in bioreactors influences the yield and quality attributes of the protein drug products. The COVID 19 pandemic revealed a vulnerability in the supply chain for necessary reagents (such as culture medium and raw material) for maintaining un-interrupted production of protein drugs with consistent quality. The supply interruption for the cell culture medium ActiPro™ optimized for producing VRC01, an IgG1-κ mAb, from a CHO-K1 cell line, necessitated the search for alternate media. VRC01 mAb is highly glycosylated and can broadly neutralize several strains of Human Immunodeficiency Virus (HIV). We investigated to see if an alternate medium can be used in the production without impacting quality attributes like glycosylation. In our strategy, we used 3 different commercially available media, performed two sets of experiments-with and without media supplements, Cell boost 7a and Cell boost 7b. Cell growth, volumetric production of the mAb protein and glycosylation pattern were compared to identify an alternative medium. Among the tested media based on cell growth, mAb production potential and glycosylation analysis, ActiCHO™ P was found to be a better alternate medium to ActiPro™ medium than EX-CELL® 325 PF CHO medium to produce VRC01 mAb. Overall, the approach used here to establish the impact of variation in medium on protein therapeutic attributes may be used during product development to build in supply chain resilience in drug manufacturing.

**Supplementary Information:**

The online version contains supplementary material available at 10.1007/s10616-025-00733-7.

## Introduction

In general, cell culture media contain essential substances such as buffering salts, amino acids, peptides, carbohydrates, vitamins, lipids and fatty acids, inorganic salts and trace elements (Yao and Asayama 2017). In addition, media also contain growth factors and hormones (Brunner et al. 2010). All these nutrients and growth supportive substances are thus made available to the cells to grow in an aqueous environment. Adaptation of the mammalian cells for biological experimentation or for production of recombinant proteins was started several decades ago. During those days the cell culture media invariably consisted of fetal bovine serum (FBS) or calf serum as a main growth supplement amounting to 5–10% (v/v) of the cell culture medium (Broedel and Papciak 2003). This is because, FBS provided the growth hormones, vitamins, fatty acids and lipids, peptides and amino acids and trace elements to enrich the other salts and buffer media which were not rich in nutrients enough to support the growth of mammalian cells (Brunner et al. 2010). However, the biological complexity, variability, and the elevated risk of contamination in the FBS media have led to batch-to-batch production variability. Furthermore, the materials of animal origin increased the risk of adventitious agents in the cell culture medium and in the drug products that were recombinantly produced using modified mammalian cell lines (Merten 1999). Subsequently, sustained efforts led to successful removal of animal derived components and serum from the cell culture media and led to cell culture media that were completely free of material of animal origin, and eventually to chemically defined media (Cui and Liu 2023; Miki and Takagi 2015; Yao and Asayama 2017). Generally, chemically defined media do not contain complex biomolecules of animal origin, which can confound the exact assessment of chemical compositions. Therefore, all chemically defined media are serum-free and pose minimal risk of contamination compared to the risk of unwanted infective agents in FBS (Usta et al. 2014).

As biotechnology improved since the 1980s, its contribution to biopharmaceuticals in health care has grown significantly and the economic significance of biotechnology industry has also become impactful (Evens and Kaitin 2015). Cell culture media development and optimization has become an important factor in biomanufacturing (Jordan et al. 2013). The traditional one-factor-at-a-time (OFAT) approach was not an ideal way to optimize the mammalian cell culture media as each medium generally contains more than 50 ingredients in composition. The aim of improving the cell line-specific medium for production (titer) and product quality became daunting as the optimization process has to take into consideration the interactions among growth, titer and product quality (Ritacco et al. 2018), and thus was labor intensive. In later days, Design of Experiments (DoE), media blending, and Process Analytical Technology (PAT) tools helped to overcome the challenges of the labor and resource intensive optimization experiments (Galbraith et al. 2018; Ritacco et al. 2018), which led to high producing product specific media development.

An optimized medium chosen for a cell line facilitates isolating and studying the impact of process variations on production and product quality of the protein therapeutic produced from the cells. During the long pandemic, an optimized medium for our cell line, an IgG1-κ monoclonal antibody producing VRC01 CHO-K1 cell line, became unavailable with protracted lead times extending beyond 56 weeks. This disruption in supply of the optimized medium necessitated re-screening of CHO culture media for our cell line. Here, VRC01 cell line was used as a model to assess the impact of process variations on production and product quality. Based on our experience with several commercially available chemically defined media that we used to evaluate production and the effect of metal compositions (Mohammad et al. 2019), we chose 3 media. Specifically, the performance of these 3 media and the effects of the additives in terms of mAb titer and glycosylation pattern of VRC01 mAb were measured to identify an alternate medium for growth and production of the VRC01 CHO-K1 cell line.

## Materials and methods

### Materials

Analytical grade chemicals and reagents were used in this study and after procurement stored at the manufacturer recommended storage temperature and conditions during investigations. EX-CELL® 325 PF CHO Serum-Free medium, L-glutamine, sodium phosphate monobasic, sodium phosphate dibasic, sodium chloride, sodium citrate monobasic and L-glycine were acquired from Sigma-Aldrich (St. Louis, MO). Other chemicals for the complete medium (sodium bicarbonate and sodium hydroxide) were purchased from Fisher Scientific (Waltham, MA). ActiCHO™ P, ActiPro™ and the additives (Cell Boost 7a and Cell Boost 7b) were purchased from Cytiva (Marlborough, MA). 10X PBS buffer was sourced from Invitrogen (Waltham, MA) and Tween® 80 from G Biosciences® (St. Louis, MO). Precast SDS-PAGE gels were from Bio-Rad (Hercules, CA) and 2X Laemmli sample buffer was from Sigma (St. Louis, MO). The buffers and enzymes used to release the N-glycans from the mAb (10X denaturing buffer, 10X glycobuffer 2, 10% NP 40, and PNGase F) were purchased from New England Bio Labs® (Ipswich, MA). Borane-2-methylpyridine complex from Alfa Aesar (Haverhill, MA) and anthranilamide and DMSO from Sigma (St. Louis, MO) were used to label the released glycans. Agencourt CleanSEQ magnetic beads were purchased from Beckman Coulter (Brea, CA), acetic acid from Fisher (Waltham, MA), and acetonitrile from VWR (Radnor, PA). Laboratory supplied deionized and filtered Milli-Q water of 18.4 MΩ resistance from a Milli-Q purification system (EMD Millipore, Burlington, MA) was used in the preparation of all aqueous buffers and reagents. 2-AB labeled N-glycan standards (G2FS2, G2FS1, G2S1, G2S2, A4G4, A3G3, A3G3S2, A3G3S1, G0, G0F, G1, G1’, G1F, G1F’, G2, G2F, Man 5, Man 6, Man 7, Man 8, and Man 9) were purchased from Agilent Technologies (Santa Clara, CA) and all glycan standards were stored at − 20 ֯C.

### Cell line

A Chinese hamster ovary origin cell line, CHO-K1 that produces an IgG1-κ mAb (VRC01) was used in our studies. This VRC01 mAb is a broadly neutralizing recombinant human monoclonal antibody against several strains of Human Immunodeficiency Virus (HIV) (Li et al. 2011; Su et al. 2014) developed by the Vaccine Research Center, NIAID, of the National Institutes of Health, Bethesda, MD, USA, that shared this cell line with FDA for regulatory science and research under a material transfer agreement.

### Shake flask culture conditions

The VRC01 CHO-K1 cells were cultured in shake flask in three media in duplicate sets (n = 2). To investigate the effectiveness of using cell culture additives (Cell boost 7a and Cell boost 7b), the culture flasks were divided into two groups. The group 1 was batch cultures using the basal media containing 8 mM L-glutamine in 500 mL baffled shake flasks with vent caps (Corning, NY). The group 2 was fed-batch cultures using the media with 4 mM L-glutamine. The lower L-glutamine concentration was compensated by daily additions of Cell boost 7a and Cell boost 7b feeds, which supplied additional amounts of L-glutamine. The additives were fed to each flask from day 2 at 2% and 0.2% of the starting culture volume for Cell boost 7a and Cell boost 7b, respectively, and maintained at 2% for Cell boost 7a and 0.2% for Cell boost 7b until harvest. A total of 12 flask cultures were included in these studies. Two independent sets for each medium for batch cultures (dp 1 and dp 2) and two other sets for fed-batch cultures (CB-dp1 and CB-dp2) added up to 12 flasks for 3 media. The VRC01 CHO-K1 cells were grown from 3 separate vials of a working cell bank in separate flasks corresponding to the 3 media, namely, ActiCHO™ P, ActiPro™ and EX-CELL® 325 PF CHO. The cells were then inoculated at the same density (~ 0.5 × 10E06 cells/ mL) for the experimentation in 3 media. The flasks in both groups were set at 125 rpm shaking speed with 5% CO_2_ mixed air circulation in an incubator (37°C). The additives Cell boost 7a and Cell boost 7b were stored at 4 °C in a refrigerator until use. One milliliter of daily samples was collected from each flask for measuring cell growth (as viable cell density, VCD, per mL) and metabolite profile, and the supernatants were collected following centrifugation (5000 × g for 5 min; Eppendorf, Hauppauge, NY) and stored frozen (-20°C) for mAb quantification. The culture was continued until the death phase of the cells in each medium to optimize the duration of culture and harvest time toward maximizing the titer level. At the end of the culture, the medium was harvested at 3000 × g for 20 min at 25°C using Sorvall Legend XTR centrifuge (ThermoFisher Scientific, Waltham, MA) and stored at -20°C until further analyses.

### Chemistry data acquisition

The nutrient trend, pH, osmolality, and partial pressures of O_2_ and CO_2_ of each flask over the cell culture period were obtained by Nova BioProfile Flex 2 (Nova Biomedical, Waltham, MA). The measured nutrients were L-glutamine (Gln, mM), L-glutamate (Glu, mM), D-glucose (Gluc, g/L), lactate (Lac, g/L), ammonium (NH4^+^, mM), potassium (K^+^, mM) and calcium (Ca^++^, mM). The frozen samples collected daily from the cultures were thawed at room temperature until no precipitation was visible and analyzed for the metabolite content.

### Measurement of mAb production profile

The mAb production in each flask was measured by bio-layer interferometry using the instrument Octet Red96e (ForteBio, Fremont, CA) on daily samples as well as harvest. The instrument utilizes protein G coated biosensors (ForteBio, Fremont, CA) that can bind with IgG antibodies. Both standards and samples were loaded (200 µL/well) to 96 well plates (Greiner Bio-One, Monroe, NC) to obtain a standard curve based on the initial rate of binding to the biosensors. Before installing the sensor tray into the instrument, the sensors were hydrated in the neutralization buffer (pH 7.4 phosphate buffered saline with tween (PBST) containing 0.05% Tween 80) for 10 min. To avoid sensor dependent bias, 3 cycles of biosensor regeneration were performed for pre-conditioning as well after each measurement. 100 mM glycine solution (pH 2.0) was used as the regeneration buffer. In addition, two sets of the standards and samples were loaded to the 96 well plate such that each set is in reverse order to the other set to facilitate exposure of the sensors to both low and high titer samples and standards. The biosensors were used for a maximum of 19 measurements and then discarded. To improve accuracy of the measurements, a pre-quantified reference standard of VRC01 mAb produced previously in the lab was used to prepare the standard set (0.078 µg/mL to 100 µg/mL). Initial binding rates were used to construct the standard curve (supplementary Fig. 1) and to determine the concentration of the mAb in the samples.

### Growth rate and production rate calculations

The specific growth rates (*µ* day^−1^) of the cultures were calculated from the day of inoculation till the end of the exponential phase (day 0 through day 4; where it showed doubling time of ~ 24 h), using the measured VCD values and the recorded time of sampling as per Eq. 1 (Hernández Rodríguez et al. 2021):

1 $$\mu = \frac{{\ln \left( {VCD_{i + 1} } \right) - {\text{ln}}\left( {VCD_{i} } \right)}}{{t_{i + 1} - t_{i} }}$$

Where ${VCD}_{i+1}$ and ${VCD}_{i}$ are the viable cell densities of the flask at the time points *t*_*i*+1_ and *t*_*i*_. Similarly, the mAb production rates (*q*, pg/cell-day) were calculated using the measured mAb concentration and the measured VCD values as per the Eq. 2 (Hernández Rodríguez et al. 2021):

2 $$q = \frac{{C_{i + 1} - C_{i} }}{{\left( {VCD_{i} + VCD_{i + 1} } \right)\left( \frac{1}{2} \right)\left( {t_{i + 1} - t_{i} } \right)}}$$

where ${C}_{i+1}$ and ${C}_{i}$ are the mAb concentrations measured in the flask at times *t*_*i*+1_ and *t*_*i*_.

### Glycan analysis by HPLC

#### Sample preparation

The mAb samples after the DEAE column purification step were used for N-linked protein glycan analysis (see supplementary material on purification methods and purity analysis). Glycans were isolated for quantification by fluorescence as previously described (Liang et al. 2024). Reagents purchased from New England Bio Labs® Inc., were used for isolating the N-glycans from the mAb samples. Twenty µL of 2 mg/mL mAb sample was used for glycan analysis. The isolated N-glycans were labeled with 2-AB following incubation at 65°C for 2.5h as previously described (Sha et al. 2020). To improve the peak resolution and signal to noise ratio, an excess dye removal method using Agencourt CleanSEQ magnetic beads (Beckman Coulter) was adopted and performed as previously described (Liang et al. 2024) before performing HPLC analysis.

#### HPLC analysis

A previously published method (Sha et al. 2020) was used with a minor modification for the analysis of cleaned 2-AB labeled N-glycan samples to accommodate more complex N-glycan profiles. HPLC analysis was performed as described previously with the same instrumentation setting and methodology (Liang et al. 2024).

### Glycan analysis by MALDI-TOF

N-glycan samples for MALDI-TOF analysis were prepared utilizing a Filter Aided N-glycan separation (FANGS) approach with materials from an Abcam Filter Aided Sample Prep (FASP) protein digestion kit (Cat#ab270519) as described (Abdul Rahman et al. 2014; Matthews et al. 2022). Briefly, 50 μg of each purified mAb sample was reduced with 200 μL urea-dithiothreitol (DTT) solution (100 mM Tris/HCL pH 8.5, containing 10 mM DTT) for 45 min while agitating the samples on a rocker operated at room temperature. The mixture was transferred to a 30 kDa molecular cutoff ultrafiltration device and centrifuged at 15,000 × g for 15 min. The mAb protein retained on the filter was washed and alkylated, as described by the manufacturer’s protocol. Finally, the mAb protein was buffer exchanged into 50 mM ammonium bicarbonate (pH 7.5), followed by 15,000 × g for 15 min, and the filter unit was transferred to a clean collection tube. One hundred μL of 50 mM ammonium bicarbonate containing 500U of glycerol-free PNGase F (Cat# P0709S, New England Biolabs, Ipswich, MA) was added to the filter, and the samples were incubated at 37 °C for 21 h. N-glycans were recovered by washing the filter with 100 μL of LC–MS-grade water followed by centrifugation at 15,000 × g for 15 min. N-glycans were then purified by active charcoal, reduced and permethylated as described (Matthews et al. 2022). Permethylated N-glycans were analyzed on a Bruker UltrafleXtreme MALDI-TOF–MS, with 2,5-dihydroxybenzoic acid (Sigma Aldrich, Cat# 149357) matrix. Mass measurements were obtained in positive ion reflector mode, and N-glycans were detected as sodiated, permethylated alditols. Monoisotopic peak intensity was converted to percent abundance based on total glycan assignments.

## Results

### Effect of additives on CHO cell growth in three media

**Fig. 1:**
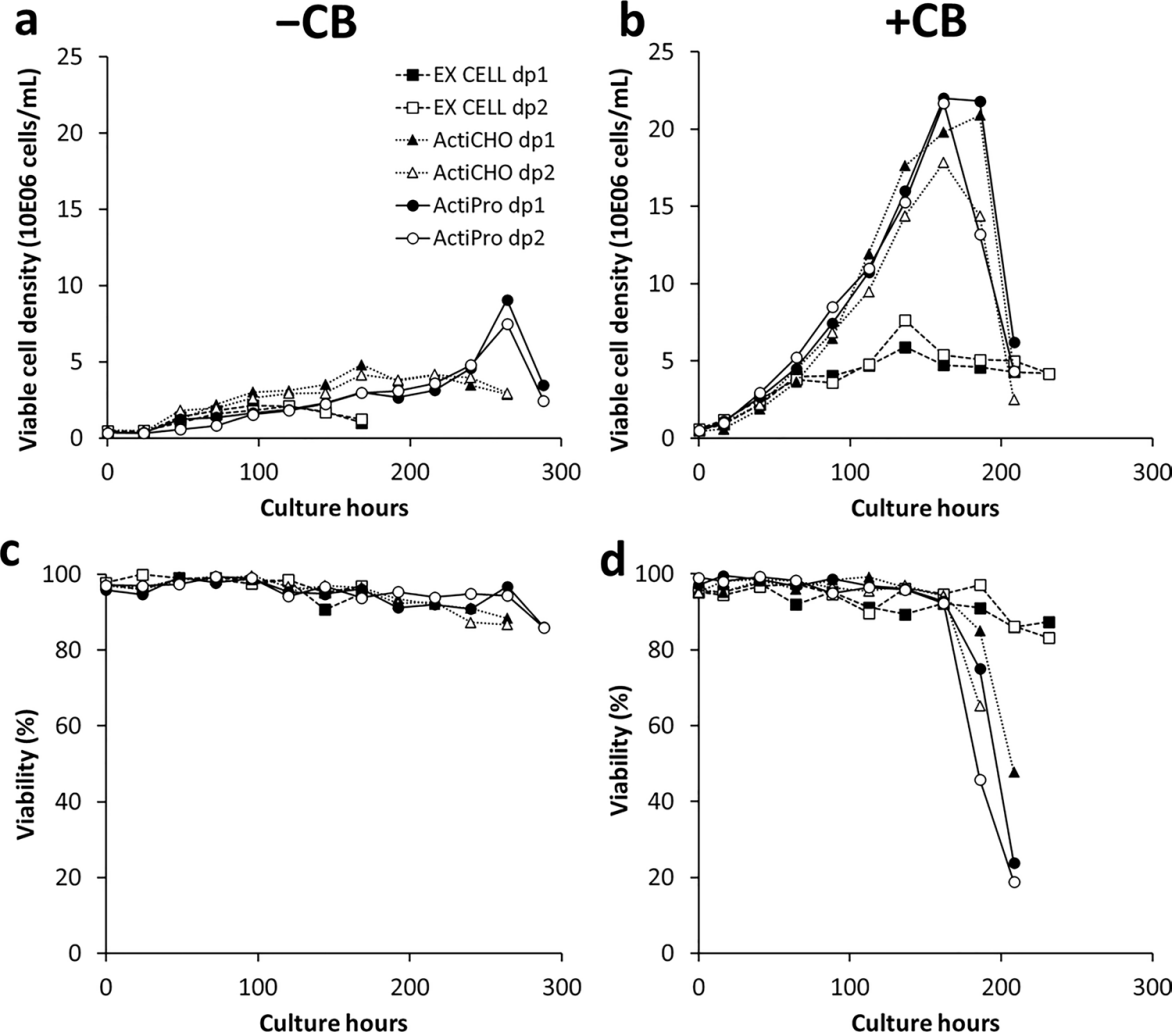
VRC01 mAb CHO-K1 cell growth and viability profiles measured during the experiments. Viable cell density for each medium is given without the additives (**a**) and with the additives (**b**). Corresponding viability (%) profiles are given in panel (**c**) for without additives and panel (**d**) for with additives

The investigation to determine compatibility of two alternate media for the VRC01 CHO cells was carried out in two groups of experiments. The group 1 experiment consisted of cell culture flasks that did not receive any growth additives or supplements. The group 2 experiment consisted of cell culture flasks that received growth additives, namely, Cell boost 7a and Cell boost 7b. Figure 1 shows the growth profiles of the VRC01 CHO cells in three media that were chosen for comparison. Growth of the VRC01 CHO cells in group 1 flasks (Fig. 1a) showed that ActiPro™ medium (8.3 × 10^6^ cells/mL) was better than the other two media (4.5 × 10^6^ cells/mL in ActiCHO™ P and 2.1 × 10^6^ cells/mL in EX-CELL® 325 PF CHO). The viability of the cells in all three media were maintained above 80% until the harvest (Fig. 1c). Growth of the VRC01 CHO cells in group 2 flasks (Fig. 1b, Table 1) showed that the maximum viable cell densities in the three media increased by 220% (for EX-CELL® 325 PF CHO, 2.1 × 10^6^ to 6.8 × 10^6^), 332% (for ActiCHO™ P, 4.5 × 10^6^ to 19.4 × 10^6^) and 163.8% (for ActiPro™, 8.3 × 10^6^ to 21.9 × 10^6^), presumably due to the additives Cell boost 7a and Cell boost 7b. The increase in growth was also reflected in growth rates (Table 1) of approximately 34, 50 and 80 percent increase for EX-CELL® 325 PF CHO, ActiCHO™ P and ActiPro™, respectively. The cell viability data showed that the cells maintained higher viability in EX-CELL® 325 PF CHO cells than in the other two media beyond 170 h of culture. Table 2 shows the differences and variations in the culture period caused by the addition of Cell boost 7a and Cell boost 7b. In group 1, the cells were cultured for 288 h in ActiPro™, 264 h in ActiCHO™ P and 168 h in EX-CELL® 325 PF CHO. The Cell boost additives appeared to shorten the culture period in ActiPro™ and ActiCHO™ P by 73.6 h and 55 h, respectively. However, the culture period of EX-CELL® 325 PF CHO was extended by 63.6 h in group 2. In each group, two replicates (dp1 & dp2) were carried out for every medium to understand consistency of response.

**Table 1:** Averaged maximum viable cell densities (n = 2) and growth rates attained without (group 1) and with (group 2) cell boost additives in the three different media

Media	Group 1	Group 2	Increase (%)
	Maximum VCD (10E06 cells/mL)		
EX-CELL 325 PF CHO	2.100	6.800	220.0
ActiCHO™ P	4.500	19.400	332.0
ActiPro™	8.300	21.900	163.8
	Growth rate (day^−1^)		
EX-CELL 325 PF CHO	0.391	0.524	33.8
ActiCHO™ P	0.465	0.704	51.2
ActiPro™	0.382	0.683	78.8

**Table 2:** Averaged (n = 2) maximum extendible duration of culture (hours) reached without (group 1) and with (group 2) cell boost additives in the three different media

Media	Group 1	Group 2	Change
EX-CELL® 325 PF CHO	168	232	+ 63.6
ActiCHO™ P	264	209	− 55.0
ActiPro™	288	214	− 73.6

**Fig. 2:**
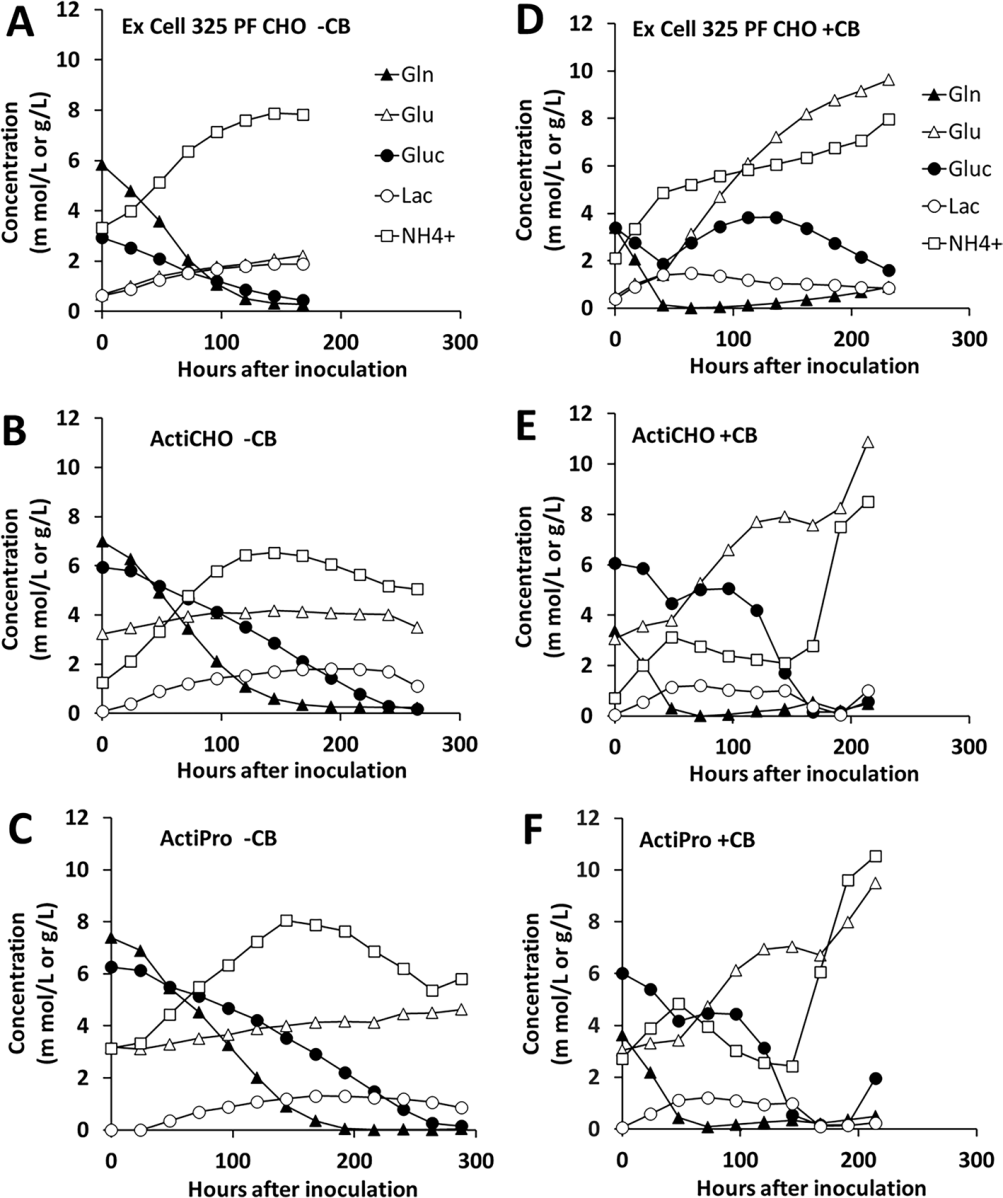
Representative profiles of metabolites in the three cell culture media. Data show the representative cultures from group 1 (absence of Cell boost additives, − CB) and group 2 (presence of Cell boost additives, + CB) for the duration of VRC01 CHO-K1 cell culture. Cell boost 7a and Cell boost 7b were used as additives. Metabolite concentrations were determined using Nova BioProfile Flex 2 instrument. Legend shown in A & D is common to all graphs

The nutrient profiles of the three media for the duration of cell culture are given in Fig. 2 with representative data for both group 1 (without additives; Fig. 2A–C) and group 2 (with Cell boost 7a and Cell boost 7b; Fig. 2D–F). Ammonium, lactic acid, and the glutamate concentrations increased progressively until approximately 100 h of cell culture, beyond which the concentrations stabilized in EX-CELL® 325 PF CHO medium (Fig. 2A). During the same period both glutamine and glucose concentrations progressively decreased and stabilized. This pattern was expected because metabolic degradation of glutamine or glutamate leads to ammonium formation and metabolic degradation of glucose leads to lactate formation. As a result, glutamine and glucose concentrations decreased progressively with culture duration (Fig. 2A–F). Surprisingly, glutamate concentration also increased, possibly due to deamination of glutamine in the medium. The cultures were started with 6 mM (in EX-CELL® 325 PF CHO) or 8 mM (in ActiCHO™ P & ActiPro™) glutamine and 3 g/L (in EX-CELL® 325 PF CHO) or 6 g/L (in ActiCHO™ P & ActiPro™) glucose in the media for the group 1, since these cultures were not added with Cell boost 7a and Cell boost 7b (Fig. 2A–C). The initial concentrations of L-glutamine and glucose were based on manufacturer’s recommendation for reconstitution of the respective media powders for preparing the initial complete media for CHO cell cultures. In group 2 cultures that were added with Cell boost 7a and Cell boost 7b, the metabolites profiles were comparable between ActiCHO™ P and ActiPro™ media (Fig. 2E and F) but different in EX-CELL® 325 PF CHO medium (Fig. 2D).

Ammonium and glutamate concentrations steadily increased in EX-CELL® 325 PF CHO medium, whereas in ActiCHO™ P and ActiPro™ media only glutamate showed somewhat steady increase with time. Ammonium on the other hand remained subdued earlier but increased rapidly after approximately 150 h of cell culture. In all three media in group 2 cultures, the lactate concentration remained less than 2 g/L throughout (Fig. 2D–F). The osmolality profiles of the duplicate sets of the experiments (dp1 & dp2) were comparable within the medium for both the groups i.e., the group without the additives (− CB) and the group with additives Cell boost 7a and Cell boost 7b (+ CB). However, a comparison among the 3 media showed that the osmolality profiles were somewhat similar between ActiCHO™ P and ActiPro™ media, whereas that of EX-CELL® 325 PF CHO medium was distinctly different (Supplementary Fig. 3).

### Effect of additives on the production of mAb in three media

**Fig. 3:**
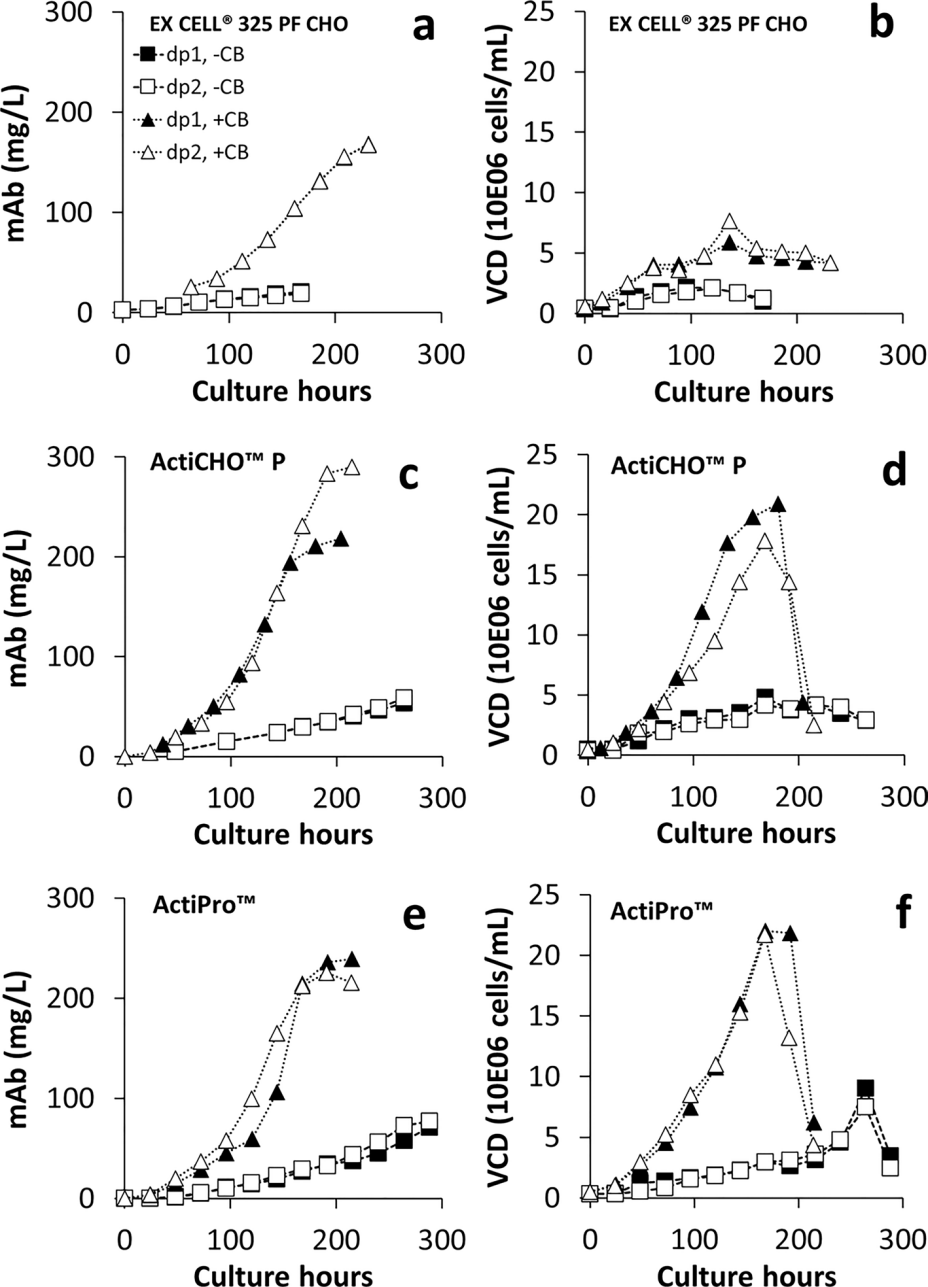
VRC01 CHO-K1 cell growth and mAb production in three media. Profiles of production and viable cell density are given for EX-CELL® 325 PF CHO (**a** and **b**), ActiCHO™ P (**c** and **d**) and ActiPro™ media (**e** and **f**). The legend shown in (**a**) is common to all other panels. In panel **c** the last three datapoints of + CB group 2 (open triangles) were outside the standard curve range and re-measured by diluting the samples. This might have contributed to a little over estimation

The production profile of VRC01 mAb from the cultures in group 1 (without additives) and group 2 (with additives Cell boost 7a and Cell boost 7b) was analyzed by taking samples daily throughout the span of cell culture period. The data are given in Fig. 3 and show the VCD data for comparison. The mAb production reached 20.1 (EX-CELL® 325 PF CHO), 56.0 (ActiCHO™ P) and 74.1 (ActiPro™) µg/mL, respectively, for the 3 media in group 1. Addition of the Cell boost 7a and Cell boost 7b in group 2 cultures improved the titer in all three media. In comparison with the group 1 cultures, the additives increased the mAb production in group 2 cultures by 735.8% (EX-CELL® 325 PF CHO, 20.1 to 168 µg/mL), 353.7% (ActiCHO™ P, 56 to 254.1 µg/mL) and 213.6% (ActiPro™, 74.1 to 232.4 µg/mL) as shown in Table 3. The effect of additives was also reflected in production rates. On average, 36, 154 and 32 percent increase in production rates were noticed, respectively, for EX-CELL® 325 PF CHO, ActiCHO™ P and ActiPro™ media (Table 3). Although the effect was maximum in EX-CELL® 325 PF CHO medium on titer improvement (~ 736%), the production of mAb per se was higher in ActiCHO™ P and ActiPro™ media (Fig. 3c and e), which appear to reflect the production rates. When compared to the maximum VCD observed in these three media, both the mAb production level and growth level were similar in ActiCHO™ P and ActiPro™ media but not in EX-CELL® 325 PF CHO medium. Thus, EX-CELL® 325 PF CHO did not perform comparably with the other two media (Table 3).

**Table 3:** Averaged (n = 2) mAb production (at harvest) and production rate without (group 1) and with (group 2) cell boost additives in the three different media

Media	Group 1	Group 2	% Increase
	Production at harvest (µg/mL)		
EX-CELL® 325 PF CHO	20.1	168.0	735.8
ActiCHO™ P	56.0	254.1	353.7
ActiPro™	74.1	232.4	213.6
	Production rate (pg/cell-day)		
EX-CELL 325 PF CHO	2.579	3.512	36.2
ActiCHO™ P	1.963	4.994	154.4
ActiPro™	3.194	4.209	31.8

### Glycosylation pattern determined by HPLC analysis

The protein-A affinity purification and the subsequent DEAE chromatography gave sufficient purity (≥ 98%) for assessing the glycosylation (Supplementary Fig. 4). Because duplicate cultures were grown from each medium for assessing consistency, duplicate sets of glycan data were obtained under the conditions of with and without Cell boost additives. We qualitatively assessed the relative intra-group consistency as well as the effect of Cell boost 7a and Cell boost 7b supplementation in group 2 cultures. Glycosylation pattern in terms of the number of quantifiable glycan types (peaks) was used as a basis for understanding the intra-group consistency. The glycosylation pattern observed from the mAb produced and purified from EX-CELL® 325 PF CHO medium was found to be less consistent in intra-group cultures either without (group 1) or with (group 2) Cell boost additives. While the glycan patterns obtained from the mAb produced in ActiPro™ medium were more consistent only in the presence of Cell boost additives, the glycan patterns of the mAb produced in the ActiCHO™ P medium were found to be more consistent intragroup, both without and with Cell boost additives (Table 4).

**Table 4:** Consistency in the glycosylation pattern and glycan content within the two sets of cell culture

Media	Consistency^a^	
	No additive	Additive
EX-CELL® 325 PF CHO	Less	Less
ActiCHO™ P	More	More
ActiPro™	Less	More

^a^Compared for the glycan peaks and their relative peak heights from HPLC analysis

**Fig. 4:**
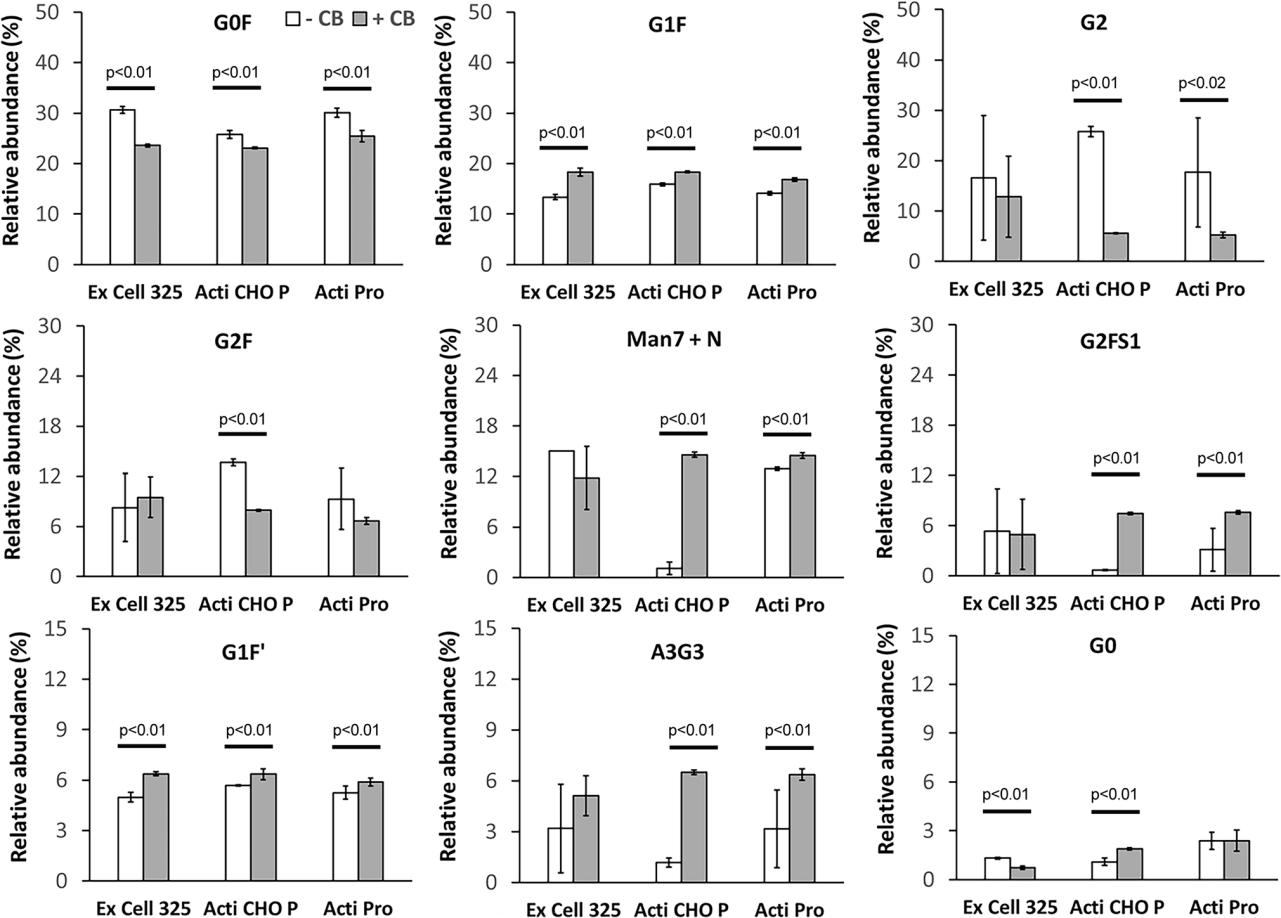
Relative abundance (% of total) of major glycan types quantified from the VRC01 mAb by HPLC analysis. The effect of Cell boost 7a and Cell boost 7b supplementation (+ CB; group 2) of the VRC01 CHO cells on various glycan type and content of VRC01 mAb was statistically compared to the un-supplemented (− CB; group 1). The significance was determined (p < 0.05) by a Dunnett’s test and only significant differences are indicated on the bars (n = 6). JMP 17.0.0 was used for statistical analysis

The major glycan types identified across all three media were G0F (22–32%), G1F (13–19%), G2 (5–26%), G2F (5–14%), Man 7 + N (1–14%), G2FS1 (2–10%), G1F’ (2–10%), A3G3 (2–10%), and G0 (2–5%). Other types of N-glycans were found to be less than 2%. Especially, those which were less than or equal to 1% were G0-N, G0F-N, Man8, Man9 and G1. The glycan types that were less than 2% were G1', Man5, Man6 and G2S1. These data can be informative to determine the effect on glycosylation of the mAb − (a) comparative effect of individual medium and (b) effect of Cell boost 7a and Cell boost 7b addition to the culture medium. Figure 4 shows the comparison of the relative abundance of the N-glycans of VRC01 mAb produced in the three media without and with the Cell boost 7a and 7b additives. Interestingly, in most cases (20 out of 27 comparisons) additives caused statistical variation in the N-glycan content of the mAb. However, comparability across the three media improved with the addition of Cell boost 7a and 7b.

Statistical analysis of the amounts of N-glycan types allowed comparability of the mAb samples produced from the three media. Table 5 shows the comparison of average amounts (percent relative abundance) of N-glycans by a Dunnett’s test performed after ANOVA on mAb samples produced without the additives. ActiPro™ medium was used as a comparator (control). The data showed that the direction and the amounts varied statistically significantly in EX-CELL® 325 PF CHO medium for more N-glycan types than those in ActiCHO™ P medium. These glycan types included G0F-N, G0, Man5, G1, G1’, G1F and Man7 + N. The Man7 + N type among others was significantly higher in EX-CELL® 325 PF CHO. In the mAb samples produced in ActiCHO™ P medium G0-N, G0F-N, G0, G0F and Man9 were significantly lower and this property was desirable (Bhide and Colley 2017; Bork et al. 2009; Falck et al. 2020) while Man5, G1F, G1F’ and Man8 were statistically higher compared to those from ActiPro™ mAb samples. Notably, Man7 + N significantly decreased in ActiCHO™ P medium.

**Table 5:** Analysis of variance (ANOVA) of mean relative abundance (%) of N-glycan types determined by HPLC analysis and comparison by Dunnett's test for the mAb samples produced without the Cell boost additives (n = 6)

Significance comparison with ActiPro™ medium (p < 0.05); Relative mean abundance (%) values with p-values ^a^					
Glycan type	F-value ^b^	p-value ^c^	ActiPro™	EX CELL® 325 PF CHO	ActiCHO™ P
G0-N	10.26	0.0016	0.60 ± 0.03	0.55 ± 0.03 (p ≤ 0.46)	0.40 ± 0.03 (p < 0.01)
G0F-N	11.01	0.0011	0.58 ± 0.04	0.40 ± 0.04 (p < 0.01)	0.35 ± 0.04 (p < 0.01)
G0	25.33	0.0001	2.38 ± 0.14	1.32 ± 0.14 (p < 0.01)	1.10 ± 0.14 (p < 0.01)
G0F	66.5	0.0001	30.07 ± 0.32	30.65 ± 0.32 (p ≤ 0.36)	25.80 ± 0.32 (p < 0.01)
Man5	10.35	0.0046	0.43 ± 0.21	1.80 ± 0.21 (p < 0.01)	1.25 ± 0.15 (p ≤ 0.02)
G1	6.23	0.0107	0.92 ± 0.05	0.68 ± 0.05 (p < 0.01)	0.80 ± 0.05 (p ≤ 0.12)
G1'	19.91	0.0001	1.47 ± 0.06	0.90 ± 0.06 (p < 0.01)	1.13 ± 0.06 (p < 0.01)
G1F	64.84	0.0001	14.13 ± 0.16	13.35 ± 0.16 (p < 0.01)	15.90 ± 0.16 (p < 0.01)
G1F'	9.12	0.0026	5.25 ± 0.12	4.98 ± 0.12 (p ≤ 0.22)	5.68 ± 0.12 (p ≤ 0.03)
Man6	2.89	0.0866	0.88 ± 0.11	0.93 ± 0.11 (p ≤ 0.93)	1.23 ± 0.11 (p ≤ 0.07)
G2	1.67	0.2215	17.68 ± 3.88	16.60 ± 3.88 (p ≤ 0.97)	25.78 ± 3.88 (p ≤ 0.27)
G2F	4.84	0.0238	9.28 ± 1.30	8.27 ± 1.30 (p ≤ 0.81)	13.65 ± 1.30 (p ≤ 0.06)
Man7 ± N	780.78	< 0.0001	12.93 ± 0.33	15.00 ± 0.33 (p ≤ 0.01)	1.05 ± 0.23 (p < 0.01)
A3G3	1.95	0.1764	3.17 ± 0.82	3.18 ± 0.82 (p ≤ 0.99)	1.18 ± 0.82 (p ≤ 0.19)
G2S1	2.45	0.1198	1.52 ± 0.14	1.10 ± 0.14 (p ≤ 0.09)	1.42 ± 0.14 (p ≤ 1.00)
Man8	20.15	0.0001	0.58 ± 0.02	0.57 ± 0.02 (p ≤ 0.85)	0.77 ± 0.02 (p < 0.01)

^a^Values in parentheses are p-values; ^b^F-values for the ANOVA; ^c^p-value for the F-statistic; ActiPro™ medium is the comparator in the Dunnett’s test, since p-values are equal to 1.00 in all instances, they are not given

From each flask 3 samples were analyzed amounting to 6 sample points per group i.e., n = 6

JMP 17.0.0 was used for statistical analysis

However, mAb samples produced from the media with Cell boost 7a and Cell boost 7b additives showed the contents of many N-glycan types that were statistically not different between the two media ActiCHO™ P and the ActiPro™ control (11 of the glycan types were statistically not different), but less so between EX-CELL® 325 PF CHO and ActiPro™ (6 of the glycan types were statistically not different) control medium. The data given in Table 6 show that G1ʹ, G1F, G1F ʹ, Man6, G2F, A3G3, and G2FS1 were not only statistically significantly different in EX-CELL® 325 PF CHO medium but also in a direction that was not favorable. The only exception was G2, which increased significantly as a desirable feature. On the other hand, only G1F and G1Fʹ were statistically different in ActiCHO™ P mAb samples, but they were increased only by less than 10%. Thus, overall, mAb produced in ActiCHO™ P medium was relatively more comparable to that produced in ActiPro™ medium in terms of glycosylation.

**Table 6:** Analysis of variance (ANOVA) of mean relative abundance (%) of N-glycan types determined by HPLC analysis and comparison by Dunnett's test for the mAb samples produced with the Cell boost additives

Significance comparison with ActiPro™ medium (p < 0.05); Relative mean abundance (%) values with p-values^a^					
Glycan type	F-value^b^	p-value^c^	ActiPro™	EX CELL® 325 PF CHO	ActiCHO™ P
G0-N	4.76	0.0250	0.33 ± 0.02	0.32 ± 0.02 (p ≤ 0.84)	0.27 ± 0.02 (p ≤ 0.02)
G0F-N	7.94	0.0044	0.41 ± 0.02	0.44 ± 0.02 (p ≤ 0.46)	0.35 ± 0.02 (p ≤ 0.03)
G0	28.74	0.0001	2.40 ± 0.16	0.74 ± 0.16 (p < 0.01)	1.89 ± 0.16 (p ≤ 0.07)
G0F	21.71	0.0001	25.45 ± 0.26	23.59 ± 0.26 (p < 0.01)	23.11 ± 0.26 (p < 0.01)
Man5	7.35	0.0082	0.75 ± 0.06	0.39 ± 0.09 (p < 0.01)	0.49 ± 0.06 (p ≤ 0.02)
G1	0.01	0.9891	0.71 ± 0.04	0.70 ± 0.04 (p ≤ 0.98)	0.71 ± 0.04 (p ≤ 0.99)
G1ʹ	12.83	0.0006	0.80 ± 0.04	0.49 ± 0.04 (p < 0.01)	0.71 ± 0.04 (p ≤ 0.25)
G1F	17.27	0.0001	16.86 ± 0.20	18.32 ± 0.20 (p < 0.01)	18.31 ± 0.20 (p < 0.01)
G1Fʹ	6.76	0.0081	5.91 ± 0.10	6.35 ± 0.10 (p < 0.01)	6.34 ± 0.10 (p < 0.01)
Man6	15.95	0.0002	0.61 ± 0.06	1.04 ± 0.06 (p < 0.01)	0.71 ± 0.06 (p ≤ 0.34)
G2	5.02	0.0214	5.30 ± 1.90	12.83 ± 1.90 (p ≤ 0.02)	5.64 ± 1.89 (p ≤ 0.99)
G2F	5.84	0.0133	6.68 ± 0.58	9.48 ± 0.58 (p < 0.01)	7.96 ± 0.58 (p ≤ 0.24)
Man7 ± N	3.08	0.0756	14.84 ± 0.89	11.83 ± 0.89 (p ≤ 0.09)	14.57 ± 0.89 (p ≤ 0.99)
A3G3	6.60	0.0088	6.36 ± 0.30	5.11 ± 0.03 (p ≤ 0.02)	6.49 ± 0.03 (p ≤ 0.93)
G2S1	196.53	< 0.0001	0.74 ± 0.02	1.47 ± 0.03 (p < 0.01)	0.66 ± 0.03 (p ≤ 0.08)
Man8				NM	NM
G2FS1	2.30	0.1339	7.59 ± 0.99	4.92 ± 0.99 (p ≤ 0.13)	7.43 ± 0.99 (p ≤ 0.99)
Man9	0.60	0.5647	0.48 ± 0.02	0.44 ± 0.02 (p ≤ 0.54)	0.48 ± 0.02 (p ≤ 1.00)

^a^Values in parentheses are p-values; ^b^F-values for the ANOVA; ^c^p-value for the F-statistic; ActiPro™ medium is the comparator in the Dunnett’s test, since p-values are equal to 1.00 in all instances, they are not given; NM, not measurable (below quantification level)

From each flask 3 samples were analyzed amounting to 6 sample points per group i.e., n = 6

JMP 17.0.0 was used for statistical analysis

### Glycosylation pattern determined by MALDI-TOF analysis

N-glycans were measured after release and permethylation by MALDI-TOF/MS on the same purified mAb samples that were used for N-glycosylation analysis by HPLC. Unlike HPLC analysis, the data showed large intra-sample variations for mAb samples produced in media without the Cell boost additives (triplicate measurements made on samples from duplicate shake flasks; n = 6). Repeating the analysis did not decrease or eliminate the intra-sample variation. Therefore, only G2F and G2S2 glycan types showed statistically different amounts when compared with the control ActiPro™ medium. The G2F type was higher in ActiCHO™ P medium and G2S2 was higher in EX-CELL® 325 PF CHO medium (data not shown). However, the mAb samples produced with the addition of Cell boost 7a and Cell boost 7b additives showed relatively less intra group (sample) variation and several glycan types showed different amounts in relative abundance that were statistically significant (Table 7). Among the 9 glycan types, 5 of them, namely, G0F, G1F, G2, G2F and G2S2 amounts were statistically significantly different in mAb samples produced from EX-CELL® 325 PF CHO than those from ActiPro™. Only G0F was statistically significantly different in ActiCHO™ P medium mAb samples. Although, G0F content decreased in both EX-CELL® 325 PF CHO and ActiCHO™ P media when compared to that produced from ActiPro™ medium, greater decrease was found in EX-CELL® 325 PF CHO. These data again indicated that mAb produced in ActiCHO™ P medium with the Cell Boost 7a and Cell boost 7b additives showed the glycosylation pattern that was more comparable to that produced from ActiPro™ medium and thus corroborated the HPLC analysis data.

**Table 7:** Analysis of variance (ANOVA) of mean relative abundance (%) of N-glycan types determined by MALDI-TOF analysis and comparison by Dunnett’s test for the mAb samples produced with the Cell boost additives

Significance comparison with ActiPro™ medium (p < 0.05); Relative mean abundance (%) values with p-values^a^					
N-glycan type	F-value^b^	p-value^c^	ActiPro™	EX CELL® 325 PF CHO	ActiCHO™ P
G0	2.7986	0.0927	1.25	0.00 (p ≤ 0.07)	0.91 (p ≤ 0.77)
G0F	50.6463	0.0001	62.22	25.50 (p < 0.01)	44.21 (p < 0.01)
G1	NS	NS	NS	NS	NS
G1F	14.5788	0.0003	30.01	21.29 (p ≤ 0.02)	36.43 (p ≤ 0.07)
G2	5.2138	0.0191	2.81	31.10 (p ≤ 0.02)	6.98 (p ≤ 0.87)
G1S1	NS	NS	NS	NS	NS
G2F	5.4464	0.0167	0.37	4.41 (p < 0.01)	2.66 (p ≤ 0.14)
G2S1	2.6532	0.1031	3.25	15.78 (p ≤ 0.07)	8.07 (p ≤ 0.60)
A3G3	NS	NS	NS	NS	NS
G2FS1	2.2145	0.1436	0.07	0.61 (p ≤ 0.13)	0.54 (p ≤ 0.20)
G2S2	3.9797	0.0411	0.00	1.28 (p ≤ 0.04)	0.17 (p ≤ 0.92)

^a^Values in parentheses are p-values; ^b^F-values for the ANOVA; ^c^p-value for the F-statistic; ActiPro™ medium is the comparator in the Dunnett’s test, since p-values are equal to 1.00 in all instances, they are not given; NS, not significant

From each flask 3 samples were analyzed amounting to 6 sample points per group i.e., n = 6

JMP 17.0.0 was used for statistical analysis

## Discussion

Process variation in the production of biopharmaceuticals can affect the product CQAs (Madhavarao et al. 2014; Parhiz et al. 2019) and changing the culture medium can affect the production yields and product quality. The composition of a chemically defined medium plays a role not only in enhancing the growth and viable cell density of the cultured cells, but also in ensuring an environment, both extracellularly and intracellularly that is physiologically congenial to produce therapeutic proteins. Furthermore, additives or supplements added to the cell culture media improve the nutritional quality of the media and boost the production of recombinant proteins from the mammalian cell cultures (Yao and Asayama 2017). However, attempts toward solely increasing production may result in unfavorable consequences on the quality parameters of the products (Costa et al. 2014; Ha et al. 2022). For example, a rigorous evaluation of doubling the production of β-glucuronidase (a lysosomal enzyme replacement therapy product) with sodium butyrate supplementation resulted in decreasing the mannose-6-phosphate content, a CQA of β-glucuronidase (Madhavarao et al. 2014). Further, Li et al., showed that any improvement in the cell culture medium composition to improve the titer of monoclonal antibodies and other therapeutic proteins also resulted in some variation in product quality attributes such as N-glycosylation (Li et al. 2010). Altering media composition even at micro-scale (micromolar concentrations) by varying the metal concentrations have also affected production and therapeutic protein quality. In this regard, metal ion composition of the media has been shown to influence the growth of cells of another CHO-K1 cell line, purity of bioreactor harvest (Graham et al. 2020) and the glycosylation (Graham et al. 2021) of the lysosomal enzyme β-glucuronidase. These findings further support that the altered medium composition can also influence production and quality of protein therapeutics. Therefore, we investigated both production and glycosylation of the VRC01 mAb to select an alternate medium.

### Growth potential

In this investigation we compared three commercially available media to produce VRC01 mAb using the CHO-K1 cell line. By performing duplicate cultures without (group 1) and with (group 2) the additives Cell boost 7a and Cell boost 7b, we could assess growth and production consistency within each medium as well as comparability across the three media. Group 1 was essentially a batch culture and group 2, a fed-batch culture. Each medium used in the group 2 cultures was reconstituted with half the concentration of L-Gln (4 mM) since the supplements Cell boost 7a and Cell boost 7b were nutrient rich products (Supplementary Table 1). In group 1 cultures, ActiPro™ performance was better than the other two media in terms of growth (higher VCD) reaching 8.3 × 10^6^ cells/mL (Fig. 1a). However, with the daily feeding of Cell boost 7a and 7b, the cells in ActiCHO™ P medium (19.4 × 10^6^ cells/mL) almost matched the level of growth achieved in ActiPro™ (21.9 × 10^6^ cells/mL) medium. Feeding the cultures with supplements was effective in increasing both the viable cell density and titer (Barrett et al. 2012; Reinhart et al. 2015). The additives increased the growth rate across all 3 media (Table 1), with a maximum increase in growth rate seen in ActiPro™ medium. However, rapid cell death was also observed in ActiCHO™ P and ActiPro™ media after the VCD reached its maximum value (Fig. 1b). This observed cell death indicated that the media were possibly depleted of some essential components (organic or inorganic nutrients) due to faster growth and higher VCD. It may be possible to overcome the fast depletion of the nutrients by selective supplementation, but this was not attempted in the current study. Thus, the cell culture periods for these two media were decreased compared to their respective batch cultures (group 1). Cell growth response in EX-CELL® 325 PF CHO was different from the other two media in group 2, showing the least increase in growth rate among the three media (Table 1). The cells remained at the stationary phase longer, and consequently, the culture period was also prolonged. However, CHO cell growth was not satisfactory in terms of VCD when compared to either ActiCHO™ P or ActiPro™ medium (Fig. 3). Increasing the cell density, even during perfusion culture, can produce microheterogeneity in terms of N-glycosylation (Liang et al. 2024). Therefore, we investigated both volumetric increase in production and glycosylation pattern in the current studies.

### Production levels of the mAb

Assessment of the production of VRC01 mAb from the CHO cells from the samples taken daily showed that in group 1, the highest concentration of VRC01 was found in ActiPro™ medium. However, in group 2, the maximum VRC01 production level of ActiCHO™ P medium (254.1 ug/mL, Fig. 2c) exceeded that in ActiPro™ (232.4 μg/mL, Fig. 2e) medium. Reinhart and coworkers had previously shown the high productivity of fed-batch process for CHO-K1 cell line with ActiCHO™ P medium and additives (Reinhart et al. 2015, 2013). We have also noticed increased production rates across all three media due to additives. The addition of Cell boost 7a and Cell boost 7b improved the performance of EX-CELL® 325 PF CHO medium (Table 3), which gave eightfold increase in titer compared to the batch cultures from the same medium (without Cell boost 7a and Cell boost 7b). Even then, the titer level was not comparable to the level produced by the ActiPro™ medium with or without the addition of Cell boost 7a and Cell boost 7b. Possibly this was due to a combination of low growth and production rates. Comparatively, ActiCHO™ P medium showed a maximum increase in production rate (~ 154%; Table 3), combined with increased growth that translated into highest titer in the presence of additives.

### N-glycosylation patterns

Among the three media, duplicate cultures were comparable in ActiCHO™ P medium with respect to glycosylation patterns in the presence of additives Cell boost 7a and Cell boost 7b and in the absence as well. The mannosylated glycan types generally decreased with the addition of Cell boost 7a and Cell boost 7b in ActiCHO™ P medium except Man7 + N type, which increased up to the level produced in ActiPro™ medium. Similarly, among the agalactosylated glycan types (G0-N, G0F-N, G0F) except for G0, all other types decreased due to the addition of Cell boost 7a and Cell boost 7b in ActiCHO™ medium. Among the galactosylated N-glycans, only G1F was somewhat increased noticeably among G1, G1ʹ, G1F and G1Fʹ type of glycans due to additives in ActiCHO™ P medium. Among the highly galactosylated glycan types (G2 and G3 containing), only G2FS1 content increased significantly due to additives in ActiCHO™ P medium and was comparable to ActiPro™ level, while other types of N-glycans either decreased in general or showed comparability. Microheterogeneity is a known risk for VRC01 mAb even when produced under perfusion cell culture when cell densities vary (Liang et al. 2024), therefore, comparison of N-glycosylation profiles was critical in the selection of an alternate medium for cell culture.

Overall, the glycosylation patterns between ActiPro™ and ActiCHO™ P media were more comparable than those produced in EX-CELL® 325 PF CHO medium (Tables 6 and 7). Taken together with the growth and mAb production levels into account, the performance of the CHO cell culture in ActiCHO™ P medium appeared to be more comparable with ActiPro™ medium and more so in the presence of Cell boost 7a and Cell boost 7b additives.

### Quality implications

Manufacturers face various scenarios under which they would have to make modifications to manufacturing process parameters or manufacturing components. Such modifications have the potential to affect production as well as product quality. Modifications mostly aimed at improving production could be adopted in manufacturing, however, the impact of such modifications on the product quality needs to be kept minimal so that the product clinical performance is unaffected. Process modifications thus effected, may improve productions and process consistency but product homogeneity may be altered. Therapeutic proteins, as they are produced from cell culture, have characteristic product profiles due to the composition of distinctly glycosylated molecules. Glycosylation being a complex critical quality attribute (CQA), it can influence product aggregation (Duran-Romaña et al. 2024), product integrity (Solá and Griebenow 2009), in vivo half-life (Falck et al. 2020), targeting (Kang et al. 2018; Solomon and Muro 2017) and efficacy (Grainger and James 2013; Wang et al. 2022; Yang et al. 2014). Therefore, studies need to be performed to confirm comparability of the product quality profile. Ensuring that the variations in microheterogeneity, arising from variation in the N-glycan type and composition, is minimal before and after the effected change in the process parameter, is essential. Furthermore, when a batch process is changed to an extended production process or continuous manufacturing process, it becomes necessary to carefully monitor the process (ICH-Q5E 2004; Liang et al. 2024). While in vivo assays are employed to assess the variation in the potency/efficacy of the therapeutic to gauge the impact on the product quality, limiting and responsible use of animals is encouraged by regulatory agencies. Therefore, strong physicochemical similarity (IEF, glycan profiles, peptide map analysis etc.) could be an indicator of comparable in vitro activities, which may substitute for in vivo assays. Only when in vitro assays cannot detect differences that could critically affect the biological function of a drug, there may be a need for in vivo assays (ICCVAM and NICEATM 2004). Keeping with the same spirit, for comparability purposes, the emphasis has shifted to physicochemical characterization, even in the case of the follow-on molecules, for establishing similarity to an approved therapeutic protein (ICH-Q5E 2004; US-FDA 2015, 2019). In our studies we have focused on evaluating the media effect on glycosylation, it being an important and formidable CQA, has been designated for analytical characterization, necessary for comparability or similarity demonstration in most of these regulatory documents.

## Conclusion

The choice of a commercially available medium for characterization of the VRC01 mAb became exceptionally important, and, based on growth and production in shake flask cultures we chose ActiPro™ medium (by Cytiva) for our investigations. However, during the unprecedented pandemic, this medium became unavailable with protracted lead times extending beyond 56 weeks. This disruption in supply necessitated re-screening of CHO culture media for our cell line. Based on experience with the previous CHO-K1 cell lines and several commercial media used for CHO cell cultures, we decided to compare two additional cell culture media that showed better performance, namely, EX-CELL® 325 PF CHO medium (Millipore Sigma) and ActiCHO™ P (HyClone medium by Cytiva) for evaluation of growth performance parameters along with ActiPro™ medium. We also investigated the effect of cell culture media additives that are commercially available (Cell Boost 7a and Cell Boost 7b from HyClone by Cytiva). The ActiCHO™ P medium gave comparable and reproducible glycosylation profiles when substituted for previously optimized ActiPro™ medium. Also, ActiCHO™ P medium resulted in a better production level compared to previously optimized conditions (ActiPro™). The addition of Cell boost 7a and Cell boost 7b to the cell culture resulted in increased viable cell densities and an increase in mAb production level and shortened the culture period by 2–3 days in ActiCHO™ P and ActiPro™ media. Our study provides a strategy to potentially prevent future disruptions in production by building redundancy in the production process during development. Such strategies will not only benefit the product and process development laboratories but can also help their manufacturing units to stay on their regular production schedule and prevent potential drug shortages.

## Supplementary Information

Below is the link to the electronic supplementary material.

Supplementary file1 (DOCX 750 KB)

## Data Availability

All data utilized to draw inferences are depicted in the form of Tables and Figures and in the supplementary material. Original source data such as HPLC runs and MALDI-TOF runs are within the institutional data repositories and can be made available when required.
