# Supplementary material for: Effect of different cell culture media on the production and glycosylation of a monoclonal antibody from a CHO cell line
Source: Cytotechnology. 2025 Mar 22;77(3):81. doi: 10.1007/s10616-025-00733-7 (PMC11928345; doi:10.1007/s10616-025-00733-7)
Supplement: Supplementary file 1 — Supplementary file1 (DOCX 750 KB) [file 10616_2025_733_MOESM1_ESM.docx]

**Supplementary material**

**MS ID: e48952f9-1430-4705-9e27-06ac681b9f94**

**Title: Production and glycosylation of a monoclonal antibody from a CHO cell line in three commercial media**

Jaeweon Lee^1,2^, Uriel Ortega-Rodriguez^3^, Chikkathur Madhavarao^1,^*, Tongzhong Ju^3^, Thomas O’Connor^1^, Ashraf Muhammad^1^, Seongkyu Yoon^2^

1, Division of Product Quality Research, OTR/OPQ/CDER, Food and Drug Administration, 10903 New Hampshire Ave, Silver Spring, MD 20993, USA

2, Department of Chemical Engineering, University of Massachusetts Lowell, Lowell, MA, USA

3, Division of Biotechnology Research and Review-III, OBP/OPQ/CDER, Food and Drug Administration, 10903 New Hampshire Ave, Silver Spring, MD 20993, USA

*Author for correspondence: [chikkathur.madhavarao@fda.hhs.gov](mailto:chikkathur.madhavarao@fda.hhs.gov)

Disclaimer: This article reflects the views of the authors and should not be construed to represent FDA’s views or policies.

**VRC01 mAb purification**

*Protein-A Affinity Chromatography*

The first purification step after harvest was protein-A affinity chromatography. A HiTrap MabSelect Prism A (Cytiva) 5 mL pre-packed column was installed on Akta avant 25 chromatography system (Cytiva). Bind and elute method was used to purify the mAb to facilitate removal of host cell proteins (HCPs) and DNA. The column was equilibrated with 20 mM sodium phosphate buffer (pH 7.0) and the bound monoclonal antibodies were eluted with 200 mM sodium citrate buffer (pH 3.0). The buffers were prepared in-house using 18.2 MΩ milli-Q water and respective salts. Crude mAb was bound to the column pre-equilibrated by 10 CV (column volumes) and the column was washed 10 CV to remove impurities. The mAb was eluted using pH 3.0 elution buffer and collected in 2.5 mL fractions in 15 mL conical-bottom tubes. The fractions containing mAbs were neutralized with 1.4 mL of 1M Tris-HCl (pH 8.5) to pH 6.0 – 6.5 and pooled in a 50 mL conical tube for further analyses.

*DEAE (diethylaminoethyl) Anion Exchange Chromatography*

The pooled elution fractions after protein-A affinity chromatography were buffer exchanged with 5 mM sodium phosphate buffer (pH 7.0) that is also used as column equilibration buffer for the DEAE column. Discontinuous diafiltration was performed by centrifugation (3600 ×g) using Amicon Ultra-15, 10,000 MWCO (Millipore Sigma, Burlington, MA) until the concentrations of the sodium citrate and Tris were minimized to 2 mM and 6 mM, respectively. The buffer exchanged sample was then purified on a 5 mL HiTrap DEAE Sepharose Fast Flow pre-packed column (Cytiva) installed on Akta avant 25 chromatography system, with 5 mM sodium phosphate buffer used in equilibrating the column. A buffer of 5 mM sodium phosphate and 200 mM NaCl was used to wash off the impurities from the column. The fractions containing mAb were pooled and concentrated to 3 – 4 mg/mL titer.

**Purity analysis by SDS-PAGE**

The relative purity levels at each step of the purification were compared using SDS-PAGE with Coomassie dye staining (Thermo Scientific, Waltham, MA). The harvested medium and the samples after each step of the chromatography were diluted with 2x Laemmli buffer (Sigma Aldrich, St. Louis, MO) and denatured at 99 °C for 10 min. The denatured samples were cooled to the room temperature and loaded on to the Criterion™ TGX™ Precast Gels (Bio-Rad, Hercules, CA). After the electrophoresis, the gel was stained and the image was captured using c600 gel imager (Azure biosystems, Dublin, CA).

**
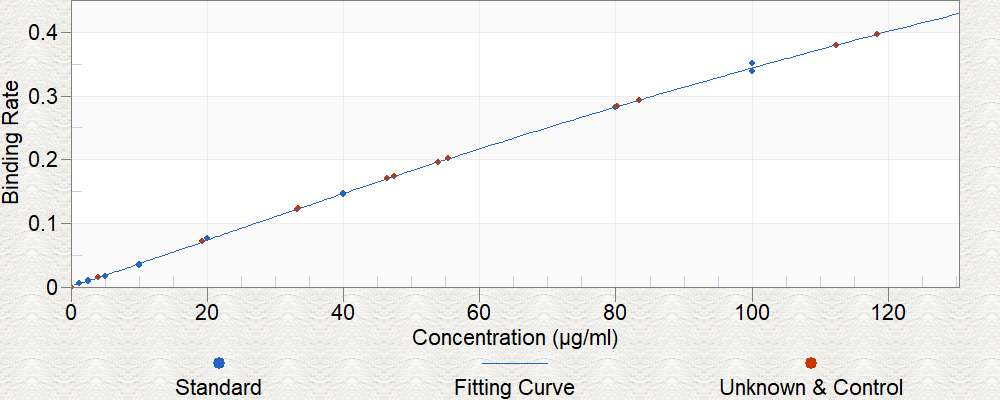
**

**Supplementary Fig. 1. Standard curve obtained for the quantification of the VRC01 mAb.**

Protein-G sensors in the Octet Red96e (ForteBio, Fremont, CA) were used to determine binding affinity of the mAb in the samples. Initial binding rates were used to obtain the standard curve.

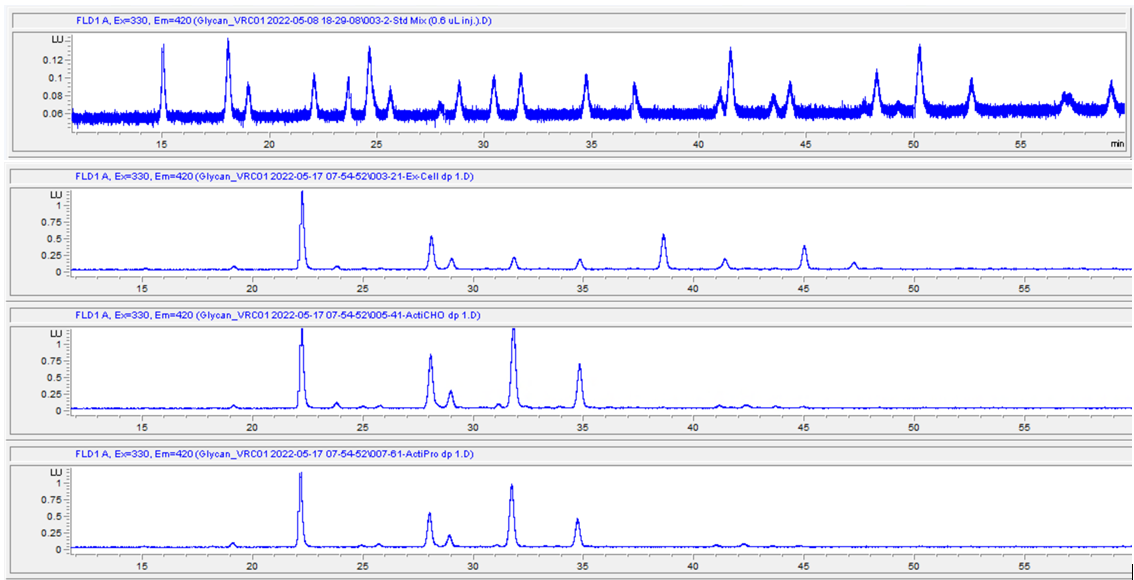


**Std.Mix**

**G0 -N**

**G0F -N**

**G0**

**G0F**

**Man 5**

**G1**

**G1’**

**G1F**

**G1F’**

**Man 6**

**G2**

**G2F**

**Man 7**

**A3G3**

**G2S1**

**Man 8**

**G2FS1**

**Man 9**

**G2S2**

**G2FS2**

**Retention Time Statistics**

**Chromatograms**

**Supplementary Fig. 2. Retention time of the N-glycan standards (top) and the HPLC elution profile of the standard N-glycan mix (bottom).**

**
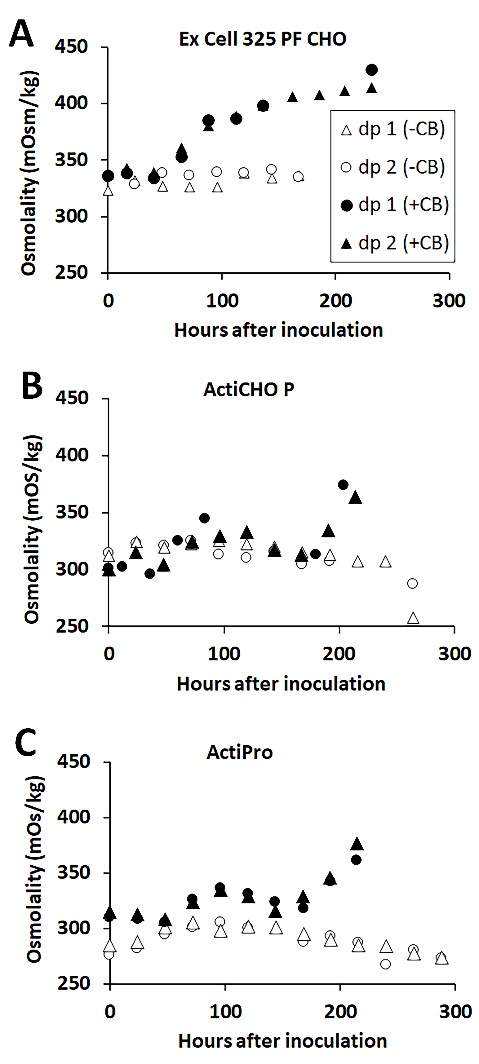
**

**Supplementary Fig 3. Osmolality profiles of the cell culture samples from the three media from start of the culture to harvest. Osmolality was measured by the BioProfile Flex 2 analyzer equipped with a freezing point depression osmometer.**

**
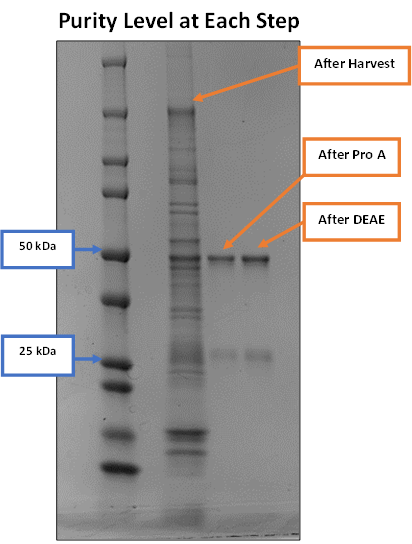
**

**Supplementary Fig. 4. Purity of the VRC01 mAb at each step of the purification is shown along with the harvest.**

**Supplementary Table 1.** The major components with approximate concentrations determined using Nova BioProfile Flex for the Cell boost additives.

|  | Concentration ^a^ | | |
| --- | --- | --- | --- |
| Products | Glutamine (mM) | Glutamate (mM) | Glucose (g/L) |
| Cell Boost 7a | 3.1 ± 0.134 | 88.8 ± 1.85 | 91.5 ± 1.29 |
| Cell Boost 7b | 8.6 ± 0.148 | 43.0 ± 3.39 | 1.1 ± 0.133 |

^a^ The values are averages of multiple measurements (n≥3)
